# Prediction of trough concentration and ALK occupancy in plasma and cerebrospinal fluid using physiologically based pharmacokinetic modeling of crizotinib, alectinib, and lorlatinib

**DOI:** 10.3389/fphar.2023.1234262

**Published:** 2023-11-22

**Authors:** Bole Li, Shan Liu, Honglei Feng, Chunshuang Du, Liman Wei, Jie Zhang, Guangwei Jia, Chunnuan Wu

**Affiliations:** ^1^ Key Laboratory of Cancer Prevention and Therapy, National Clinical Research Center for Cancer, Tianjin’s Clinical Research Center for Cancer, Tianjin Medical University Cancer Institute and Hospital, Tianjin, China; ^2^ Department of pharmacy, Affiliated Hospital of Shandong University of Traditional Chinese Medicine, Jinan, Shandong, China; ^3^ Key Laboratory of Clinical Pharmacology Liaocheng People’s Hospital, Liaocheng, Shandong, China

**Keywords:** ALK inhibitors, PBPK model, ALK occupancy, optimal dosing regimen, concentration prediction in cerebrospinal fluid

## Abstract

**Backgrounds:** Brain metastases occur in approximately 30% of patients with non-small-cell lung cancer (NSCLC). Therefore, the free drug concentration in cerebrospinal fluid (CSF) is strongly associated with the clinical efficacy.

**Purpose:** The present study aimed to develop physiologically based pharmacokinetic (PBPK) models that can predict the steady-state trough concentration (C_trough_) in plasma and CSF, as well as anaplastic lymphoma kinase (ALK) occupancy (AO), for three inhibitors: crizotinib (CRI), alectinib (ALE), and lorlatinib (LOR).

**Methods:** To achieve this, population PBPK models were successfully developed and validated using multiple clinical pharmacokinetics (PK) and drug–drug interaction (DDI) studies, both in healthy subjects and patients.

**Results:** The prediction-to-observation ratios for plasma AUC, C_max_, and C_trough_ in heathy subjects and patients ranged between 0.5 and 2.0. In addition, PK profiles of CRI, ALE, and LOR in CSF aligned well with observed data. Moreover, the AUC and C_max_ ratios of the three inhibitors when co-administered with CYP3A4 inhibitors/inducers also matched with clinically observed values. Utilizing PK thresholds for effective plasma C_trough_ and AO values on wild-type and four ALK mutations in plasma and CSF, PBPK models were then combined with the mean and 95% confidence interval to predict optimal dosing regimens.

**Conclusions:** Overall, these PBPK models provide valuable insights into determining appropriate dosing regimens for the three ALK inhibitors, understanding their effectiveness in brain metastasis therapy, and analyzing the underlying mechanisms of on-target resistance.

## 1 Introduction

Lung cancer is the leading cause of global cancer-related deaths, accounting for approximately 18.4% of all cancer mortality worldwide in 2018 (Thandra et al., 2021). Non-small-cell lung cancer (NSCLC) constitutes more than 80% of all lung cancer cases (Fujimoto et al., 2019). The anaplastic lymphoma kinase (ALK) gene was first identified as a lung oncogene in 2007 (Lei et al., 2022). The reported incidence of ALK-positive NSCLC ranges from 3% to 7% (Gower et al., 2020). A clinical study has demonstrated that cancer cells carrying an ALK rearrangement (ALK-positive) are sensitive to ALK inhibition (Shaw et al., 2011). In addition, brain metastases have been reported to occur in approximately 30%–40% of ALK-positive NSCLC patients (Zou et al., 2022).

Crizotinib (CRI) is a first-generation ALK inhibitor that received the FDA approval in 2011 for the treatment of ALK-positive NSCLC (OBryant et al., 2013). The recommended dosage for patients is 250 mg orally twice daily (BID) (Food and Drug Administration FDA, 2011a). CRI is predominantly metabolized by cytochrome P450 (CYP3A4), accounting for 99.4% based on findings from a recombinant expressed CYP isoform experiment (Food and Drug Administration FDA, 2011a). PF-06260182 is the only identified metabolite accounting for more than 10% *in vivo* (Food and Drug Administration FDA, 2011a). However, in contrast to CRI, PF-06260182 exhibits approximately 3- to 8-fold less potency against ALK (Food and Drug Administration FDA, 2011a). Furthermore, CRI binds to human plasma albumin to a degree of 91% (Food and Drug Administration FDA, 2011a). Studies have shown a low penetration rate of CRI into the cerebrospinal fluid (CSF) in humans (Metro et al., 2016). Alectinib (ALE) is a second-generation ALK inhibitor and the first one approved in 2017 for the therapy of ALK-positive NSCLC with brain metastases (Herden and Waller, 2018). The recommended dosage is 600 mg orally twice daily (BID) (Herden and Waller, 2018). ALE is primarily metabolized by CYP3A4 to its active metabolite M4, which accounts for approximately 40% of ALE metabolism (Food and Drug Administration FDA, 2011b). M4 exhibits potent activity against human recombinant ALK, with an IC_50_ value comparable to ALE (Food and Drug Administration FDA, 2011b). Both ALE and M4 are bound to human plasma albumin by more than 99%, regardless of their concentrations (Food and Drug Administration FDA, 2011b). Although ALE has a good ability to cross the blood–brain barriers, as evidenced by an unbound CSF to unbound plasma ratio of 20%–50% (Food and Drug Administration FDA, 2011b), studies have shown that it has low penetration into the CSF. The unbound CSF concentrations of ALE range from 0.2% to 0.5% of the total ALE concentration in the plasma. Lorlatinib (LOR) is the third-generation ALK inhibitor and was approved in 2018 for the treatment of patients with ALK-positive metastatic NSCLS (Syed, 2019). The *in vitro* experiment showed that LOR is mainly metabolized by CYP3A4 and UGT1A4, with minor contributions from CYP3A5, CYP2C8, CYP2C19, and UGT1A3 (Food and Drug Administration FDA, 2011c). Metabolite M8 accounts for 21% of human plasma radioactivity (Food and Drug Administration FDA, 2011c). However, M8 is pharmacologically inactive (Food and Drug Administration FDA, 2011c). LOR exhibits moderate binding to serum albumin and α1–acid glycoprotein, with a plasma protein binding of 66% (Food and Drug Administration FDA, 2011c). LOR demonstrated high penetration into CSF, with concentrations as high as approximately 75% of those in plasma (Singh and Chen, 2020).

To date, multiple types of ALK mutations have been identified. Of these ALK mutations, ALKL^1196M^, ALKG^1269A^, and ALK^G1202R^ are the most common mutations in patients, and ALK^G1202R^ confers high-level resistance to almost all of the ALK inhibitors (Shaw et al., 2017). The brain was the most common single site of disease progression after CRI treatment (Shaw et al., 2017). For an ALK inhibitor to be effective, it must cross the blood–brain barrier to reach target cell with sufficient free concentration. Therefore, the efficacy of ALK inhibitors in addressing brain metastasis development in NSCLC patients is influenced by two potential significant factors: activity against ALK mutations and a high penetration rate into CSF.

For continuous dosing of medications to be effective, maintaining a sufficient plasma trough concentration (C_trough_) at the steady state is crucial for optimal clinical efficacy. Clinical studies have established C_trough_ thresholds for certain drugs: ≥1,000 ng/mL for imatinib (Picard et al., 2007) and ≥32 μg/mL for pazopanib (Wu et al., 2022). These thresholds are associated with favorable clinical outcomes. In addition, the level of kinase occupancy has been shown to strongly correlate with the overall response rate (ORR). For example, a clinical study demonstrated that achieving >90% Bruton’s tyrosine kinase (BTK) occupancy by acalabrutinib resulted in an ORR exceeding 80% (Food and Drug Administration FDA, 2011d). Similarly, another study found that achieving at least >75% ALK occupancy by CRI is necessary for clinically effective treatment. Therefore, the plasma C_trough_ level and ALK occupancy play important roles in determining the clinical efficacy. Furthermore, high C_trough_ levels in CSF and significant intracranial ALK occupancy can suggest greater effectiveness in the clinical therapy of brain metastases in NSCLC patients.

Physiologically based pharmacokinetic (PBPK) modeling is a promising tool to predict the C_trough_ at the steady state in human plasma and CSF. This approach has been extensively used to predict human plasma and tissue concentrations (Yamamoto et al., 2017; Adiwidjaja et al., 2022) as well as the target occupancy (Xu et al., 2022). However, the current PBPK models lack the ability to directly simulate the concentration in CSF. As an alternative, the free concentration in the interstitial fluid of brain tissue is simulated to approximate the concentration in CSF. The main objectives of the present work are as follows:

(i) To develop PBPK models for CRI, ALE, and LOR in both healthy individuals and cancer patients.
(ii) To develop ALK occupancy models in plasma and CSF for CRI, ALE, and LOR.
(iii) To simulate C_trough_ and ALK occupancy at the steady state in plasma and CSF and predict drug–disease interaction outcomes for brain metastasis patients.

## 2 Materials and methods

### 2.1 Physiologically based pharmacokinetic model development

#### 2.1.1 Healthy physiologically based pharmacokinetic model

Whole-body PBPK models were developed using PK-Sim^®^ (version 11.1, Bayer Technology Services, Leverkusen, Germany) for three ALK inhibitors (CRI, ALE, and LOR) in healthy subjects. The developed PBPK models were utilized to retrospectively analyze the PK data of CRI, ALE, and LOR in healthy individuals.

The PBPK model is constructed by connecting tissue compartments using the blood flow rate. It includes essential components such as the gastrointestine, arterial supply, and venous return of blood. Tissues with elimination functions, like the liver and kidney, are included, whereas non-eliminating tissues like the lung are also considered. To account for the transfer of drugs between compartments, the model incorporates permeability-limited perfusion, which assumes that the distribution of a drug within each tissue is primarily governed by its permeability across the tissue barriers. The Weibull times of ALE and LOR were optimized using *in vitro* dissolution profiles from the study (Food and Drug Administration FDA, 2011c; Kato et al., 2020). The Weibull time of CRI was optimized using the PK-Sim method based on its plasma concentration–time profiles. Human tissue distribution was described using Rodgers and Rowland’s methods, whereas cellular permeability was determined using the standard PK-Sim method. The K_IA_ scale (intracellular space-to-plasma partition) was optimized to values of 5.0, 2.0, and 3.0 for the three inhibitors to better describe their distribution based on their respective PK profiles and distribution volumes. The PK-Sim model divided the brain tissue compartment into four sub-compartments: plasma, blood cells, interstitial, and intracellular space. The distribution across the capillary membrane is assumed to incorporate permeability-limited perfusion. In a previous PBPK study (Diestelhorst et al., 2013), the concentration in the interstitial sub-compartment was assumed to represent that in the CSF. In this study, to predict the free concentration of three inhibitors in the CSF, the unbound concentration in interstitial fluid is also assumed to be equal to the free concentration in the CSF. In addition, the K_IR_ scale (interstitial space-to-plasma partition) was optimized to be a value of 2.0 for ALE, whereas CRI and LOR were assigned a value of 1.0 for this parameter.

The clearance of ALK inhibitors primarily occurs through hepatic metabolism, and most intrinsic clearance (CL_int_) parameters were obtained from references (see Table 1). In the case of CRI, its efflux transport is described by its intrinsic transport velocity (CL_int_). The CL_int_ P-gp (P-glycoprotein) value for CRI was estimated to be 1.9 μL/min/million cells^-1^ based on the P_eff_ (effective permeability) data from transfected MDCKII with huABCB1 (Tang et al., 2014). The total hepatic clearance (CL) of ALE was estimated to be 34.5 L/h (Morcos et al., 2017a). In addition, the additional plasma clearance (CL_a_) was calculated to be 0.28 L/h/kg using the formula derived by Simulations-Plus (2019). For LOR, CL_int_ is scaled using Eq. 1:

$${\text{CL}}_{\mathrm{int}}=\text{MV}\times \text{ISEF},$$ (1)

**TABLE 1:** Physiologically based pharmacokinetic input parameters of crizotinib, alectinib, and lorlatinib used in the simulations using PK-Sim.

Property (Unit)	CRI		ALE		M4	LOR	
MW (g·mol^-1^)	450.3		482.6		456.6	406.4	
Basic pKa	5.6, 9.4 (Food and Drug Administration FDA, 2011a)		7.2 (Alsmadi et al., 2021)		7.35 (Food and Drug Administration FDA, 2011b)	4.9 (Food and Drug Administration FDA, 2011c)	
Log P	4.28 (Food and Drug Administration FDA, 2011a)		4.69 (Alsmadi et al., 2021)		4.69 (assigned)	2.47 (Food and Drug Administration FDA, 2011c)	
Solubility (mg·mL^-1^)	0.74 (pH6.5) (Christina Fink et al., 2020)		0.023 (FaSSIF, pH6.5) (Alsmadi et al., 2021)		-	0.11 (Damoiseaux et al., 2022)	
Intestinal permeability (cm⋅s^-1^)	P_app_:16✕10^−6^ (Di et al., 2020)		P_app_ 1.88✕10^−6^ (Alsmadi et al., 2021)		-	P_app_: 28✕10^−6^ (Food and Drug Administration FDA, 2011c)	
f_up_/f_up_′^a^	0.093/0.13 (Food and Drug Administration FDA, 2011a)		0.003/0.004 (Food and Drug Administration FDA, 2011b)		0.006/0.009 (Food and Drug Administration FDA, 2011b)	0.34/0.43 (Food and Drug Administration FDA, 2011c)	
R_bp_/R_bp_’^a^	1.1/1.2 (Food and Drug Administration FDA, 2011a)		2.6/3.0 (Food and Drug Administration FDA, 2011b)		2.5/2.8 (Food and Drug Administration FDA, 2011b)	0.99/1.1 (Food and Drug Administration FDA, 2011c)	
CL_R_ (L/h)	GFR*f_up_						
GFR fraction	1.0 (default)						
K_IA_ scale	5.0 (optimized)		2.0 (optimized)		0.5 (optimized)	3.0 (optimized)	
K_IR_ scale	1.0		2.0 (optimized)		0.5 (optimized)	1.0	
Weibull time (min)	45 (optimized)		60 (optimized)		-	30 (optimized)	
Weibull shape	0.92 (default)						
**Metabolic parameters**							
	CL_int_3A4 (μL/min/mg protein)	103 (Yamazaki et al., 2015)	CL_int_3A4 (μL/min/pmol)	9.98 (Food and Drug Administration FDA, 2011b)	1.71 (Food and Drug Administration FDA, 2011b)	Converting to M6 (calculated)	
	CL_int_ P-gp (μL/min/million cells^-1^)	1.49 (calculated)^[24]^	Unspecified HLM CL_int_ (μL/min/mg protein)	1710 (Food and Drug Administration FDA, 2011b)	1330 (Food and Drug Administration FDA, 2011b)	CYP3A4 V_max_ (pmol/min/mg)	3.10 (Food and Drug Administration FDA, 2011c)
	P-gp K_m_ (μM)	8.5 (optimized)	CL_a_ (L/h/kg)	0.28 (calculated)	0.42 (optimized)	CYP3A4 K_m_ (μM)	2.12 (Food and Drug Administration FDA, 2011c)
						Converting to M2a, CL_int_ (μL/min/mg)	
						CYP3A4	0.042
						CYP3A5	0.11
						CYP2C8	0.20
						CYP2C19	0.05
						Converting to M1a, CL_int_ (μL/min/mg)	
						UGT1A3	0.012
						UGT1A4	0.10
**Concentration (μM/L liver tissue)**	Metabolism enzymes	Default: CYP3A4/CYP3A4′^a^:4.32/3.02; CYP3A5:0.04; CYP2C8:2.56; CYP2C19/CYP2C19′^a^:0.76/0.51 (calculated); UGT1A3/A4:0.53/0.25 (calculated)					
	Transporters	Calculated: P-gp: 0.68					
**Interactions**							
K_i_ CYP3A4 (μM)	1.9 (mean value) (Food and Drug Administration FDA, 2011a; Yamazaki et al., 2015)		8.3 (Food and Drug Administration FDA, 2011b)		7.0 (Food and Drug Administration FDA, 2011b)	328.2 (Food and Drug Administration FDA, 2011c)	
k_inact_ CYP3A4 (h^-1^)	6.6 (Food and Drug Administration FDA, 2011a)		3.7 (Food and Drug Administration FDA, 2011b)		3.7 (Food and Drug Administration FDA, 2011b)	-	
K_i_ P-gp (μM)	7.8 (Eliesen et al., 2017)		-		-	-	
EC_50_ CYP3A4 (μM)	0.24 (optimized) (Yamazaki et al., 2015)		1.0 (optimized) (Food and Drug Administration FDA, 2011b)			0.29 (Food and Drug Administration FDA, 2011c)	
E_max_ CYP3A4	10.4 (optimized)		3.5 (optimized) (Food and Drug Administration FDA, 2011b)			5.99 (Food and Drug Administration FDA, 2011c)	

^a^ Values in healthy subjects and NSCLC patients, respectively.

-, no data; MW, molecular weight; basic pKa, base dissociation constant; log P, lipophilicity; f_up_, free fraction in plasma; R_bp_, blood-to-plasma concentration ratio; CL_R_, renal clearance; GFR fraction, fraction of filtered drug in the urine; GFR, glomerular filtration rate; K_IA_, intracellular space-to-plasma partition; K_IR_, interstitial space-to-plasma partition; CL_a_, additional plasma clearance; Weibull time, dissolution time of 50% drug; Weibull shape, shape parameter of Weibull function; CL_int_ 3A4, intrinsic clearance for CYP3A4; HLM CL_int,u_, intrinsic clearance for human liver microsome; CL_int_ P-gp, transport rate by P-gp; V_max_, maximum metabolism velocity; K_m_, Michaelis–Menten; K_i_, 50% maximal inactivation rate; k_inact_, maximum rate of inactivation; EC_50_, inducer concentration required to achieve 50% inductive effect; E_max_, maximum inductive effect for CYP3A4.

where MV is the original metabolic velocity (pmol/min/pmol enzyme), ISEF represents intersystem extrapolation factor, and ISEF values were used at 0.21 for CYP3A4, 0.12 for CYP3A5, 1.41 for CYP2C8, 0.25 for CYP2C19, and 0.077 for UGT1A3 and UGT1A4 according to the literature works (Food and Drug Administration FDA, 2011c; Conner et al., 2019).

In three PBPK models, six metabolizing enzymes (see Table 1) and one transporter (P-gp) were included. The reference concentrations of UGT1A3/1A4 and P-gp have not been integrated into the PK-Sim expression database. In PK-Sim, the reference concentration of CYP enzymes or transporters is represented as the concentration per unit volume in the liver (μM/L). Therefore, the reference concentration of UGT1A3/1A4 in the liver was calculated by Eq. 2:

$$\left.\text{UGT}1A3/4\text{ concentration}=\text{UGT}1A3/4\text{ abundance}✕\text{mg CYP protein}/\left.g\text{ liver}\right)/\text{liver volume}\right),$$ (2)

where UGT1A3/4 abundance (pmol/mg protein) values were assigned to be 15.3 and 44.3 from the study by Reddy et al. (2021) and protein abundance (mg protein/g liver) was set at 45.0 according to the study by Qian et al. (2019). The default values of liver weight and volume in PK-Sim were used.

The expression level of P-gp in brain tissue was determined based on the relative expression percentage. Reference concentration of P-gp in the liver is calculated by Eq. 3:

P‐gpconcentration=P‐gpabundance✕organ weight✕Ratio/liver volume. (3)

In the current paper (Couto et al., 2020), the abundance of P-gp was experimentally determined only in the human intestine, where it was found to be 1.60 pmol/mg tissue. Consequently, the weight of the intestinal tract was used as an approximation for the reference concentration of P-gp in the liver. The ratio is 0.56 (relative expression ratio of liver-to-intestine). By considering the reference concentration and relative expression in the brain, the expression of P-gp in brain tissue is then converted accordingly. Moreover, based on the literature works, CYP abundances in PK-Sim were set at 137, 103, 24, 14, 15, and 44 pmol/mg protein for CYP3A4, 3A5, 2C8, 2C19 (Food and Drug Administration FDA, 2011c), UGT1A3, and UGT1A4 (Reddy et al., 2021), respectively.

#### 2.1.2 Diseased physiologically based pharmacokinetic model

In the diseased PBPK model, the overall structure remains the same as the healthy PBPK model described earlier. However, certain modeling parameters were adjusted based on relevant published articles for populations with cancer. The specific changes are as follows: ➀downregulation of hepatic CYP3A4 and CYP2C19 expression levels: in cancer patients, the expression level of hepatic CYP3A4 is reduced by 45% compared to the healthy population. Similarly, the expression level of hepatic CYP2C19 is reduced by 30%. The corresponding values for these downregulated expression levels are reported as 3.02 μM/L liver tissue for CYP3A4 and 0.51 μM/L liver tissue for CYP2C19 (Schwenger et al., 2018) (see Table 1). ➁Reduced patients’ plasma albumin level (g/dl): hematocrit: cancer patients exhibit decreased levels of plasma albumin (from 4.5 g/dl in healthy individuals to 3.1 g/dl) and hematocrit (from 0.43 to 0.33) (Dixon et al., 2003). ➂Overexpression of P-gp: in patients with resistance to CRI, an overexpression of P-gp has been observed in patient-derived cells (Li et al., 2018). To simulate this effect, the concentration of P-gp at the blood–brain barrier in the brain in CRI simulations was set two-fold higher than that in the healthy PBPK model. However, in the PBPK model, it is not possible to directly set the concentration of P-gp at the blood–brain barrier. Instead, as an alternative approach, data from the brain were utilized to assign the concentration of P-gp for CRI simulations. Furthermore, the scaling of f_up_ and R_bp_ in cancer patients was performed using the following Eqs 4–6 (Trevor et al., 2010; Simulations-Plus, 2019):

$$f_{\text{up}}^{\prime }=1/\left(1+\left(\left(1‐f_{\text{up}}\right)✕{\left[P\right]}^{\prime }\right)/\left(\left[P\right]✕f_{\text{up}}\right)\right),$$ (4)

where f_up_’ and f_up_ are free plasma fractions in patients and healthy subjects, respectively, and [P]′ and [P] are the plasma albumin protein concentrations in patients and healthy subjects, respectively.

$$R_{\text{bp}}^{\prime }=1+\text{Hct}✕\left(f_{\text{up}}*K_{\text{puBC}}‐1\right),$$ (5)

where R_bp_’ is the blood-to-plasma concentration ratio in patients, Hct is the hematocrit value, and K_puBC_ is the affinity of blood cells to the drug. K_puBC_ was calculated as follows:

$$K_{\text{puBC}}=\left(\text{Hct}‐1+\text{Rbp}\right)/\left(\text{Hct}✕f_{\text{up}}\right).$$ (6)

**FIGURE 1:**
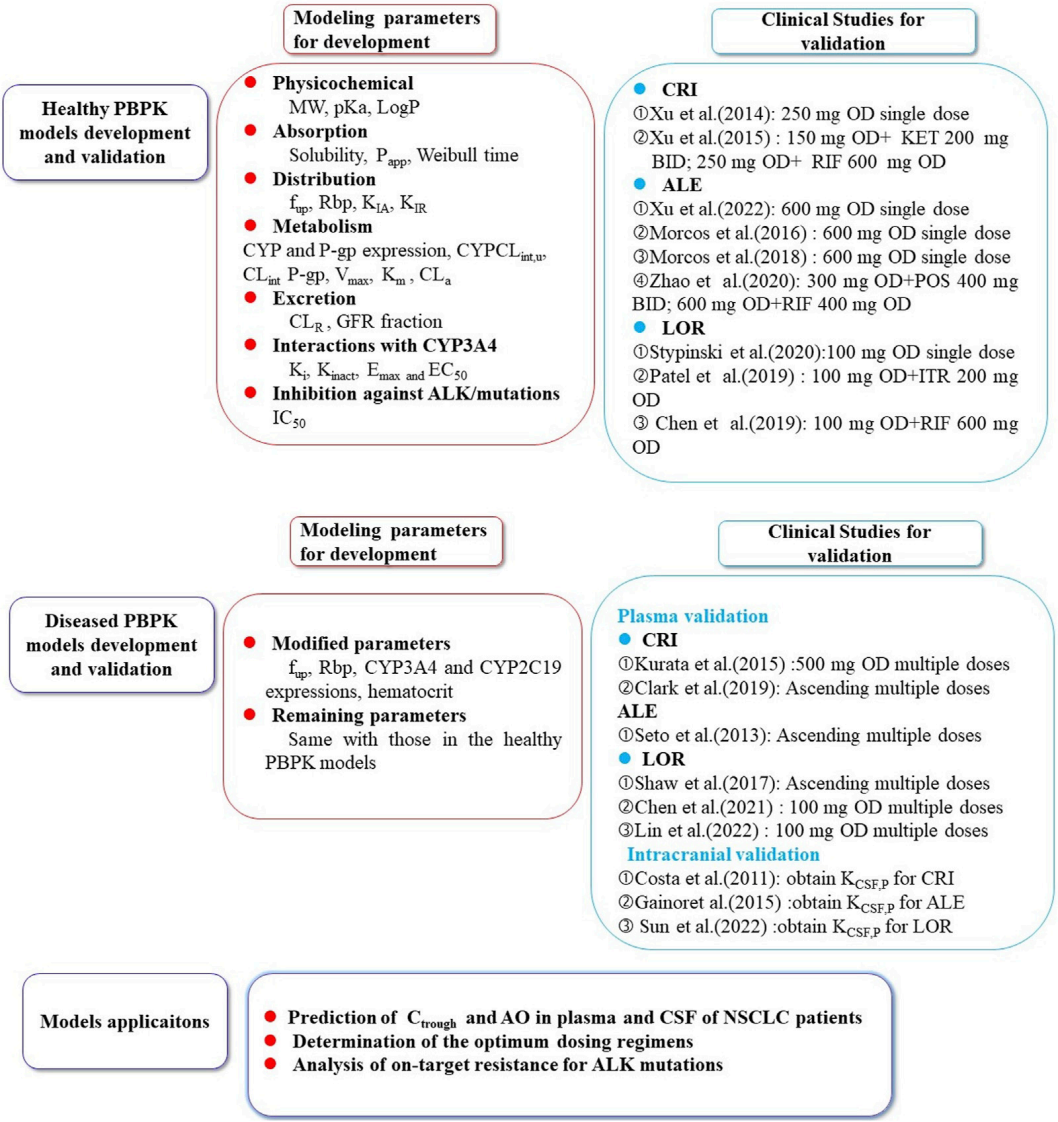
The schematic representation of the PBPK models for crizotinib, alectinib, and lorlatinib. The population PBPK models of the three ALK inhibitors were built based on the physicochemical parameters, absorption, distribution, metabolism, excretion, and interaction processes involved in CYP3A4/5, 2C8, 2C19, UGT1A3/4 metabolizing enzymes and P-gp transporter. The models were validated using multiple PK data in healthy subjects and NSCLC patients (Seto et al., 2013; Gadgeel et al., 2014; Xu et al., 2015a; Kurata et al., 2015; Morcos et al., 2017a; Morcos et al., 2017b; Morcos et al., 2017c; Shaw et al., 2017; Clark et al., 2019; Stypinski et al., 2020; Chen et al., 2021; Hibma et al., 2022; Huiping et al., 2022; Lin et al., 2022). CRI: crizotinib; ALE:alectinib; LOR: lorlatinib; KET: ketoconazole; RIF: rifampin POS: posaconazole; ITR: itraconazole; CSF: cerebrospinal fluid; K_CSF,P_: CFS-to-plasma ratio; C_trough_: trough concentration; AO: ALK occupancy; NSCLC: non-small cell lung cancer.

The remaining modeling parameters for the three inhibitors were assumed to be identical to healthy conditions. The complete set of parameters for the model is summarized in Table 1 (Food and Drug Administration FDA, 2011a; Food and Drug Administration FDA, 2011b; Food and Drug Administration FDA, 2011c; Tang et al., 2014; Yamazaki et al., 2015; Eliesen et al., 2017; Christina Fink et al., 2020; Di et al., 2020; Alsmadi et al., 2021; Damoiseaux et al., 2022). The schematic representation of the PBPK models can be observed in Figure 1.

### 2.2 Physiologically based pharmacokinetic model verification and prediction evaluation

#### 2.2.1 Verification using PK profiles and data

To validate the predictive performance of the PBPK model, multiple clinical PK profiles for the three inhibitors were used. These profiles included data from both healthy subjects and patients. The validation process involved comparing the coincidence of predicted PK profiles with the observed ones. In addition, the models were verified by comparing the ratios between the predicted and observed AUC, C_max_, and C_trough_ (Seto et al., 2013; Gadgeel et al., 2014; Xu et al., 2015a; Kurata et al., 2015; Morcos et al., 2017a; Morcos et al., 2017b; Morcos et al., 2017c; Shaw et al., 2017; Clark et al., 2019; Stypinski et al., 2020; Chen et al., 2021; Hibma et al., 2022; Huiping et al., 2022; Lin et al., 2022) following single dose and repeated doses. Furthermore, the models were further validated by comparing predicted and calculated PK profiles in the CSF. Drug concentration (C_CSF(t)_) was calculated by the following equation:

$$C_{\text{CSF}\left(t\right)}=C_{p\left(t\right)}✕f_{\text{up}}^{\prime }✕\;K_{\text{CSF},P},$$ (7)

where C_p(t)_is the plasma concentration in the vein at different time points and K_CSF,P_ is the CSF-to-plasma ratio. For the three ALK inhibitors, K_CSF,*p*_ values were obtained from the studies by Costa et al. (2011), Gainor et al. (2016), and Sun et al. (2022). The assigned values for the three ALK inhibitors were calculated to be 0.0026, 0.79 (mean value), and 0.77, respectively.

#### 2.2.2 Verification using drug–drug interaction simulations

In order to ensure the contribution of CYP3A4 to total clearance and the accuracy of inhibition and induction parameters of CYP3A4, multiple drug–drug interaction (DDI) simulations were conducted. First, the PK effects of CRI and LOR on midazolam (CYP3A4 substrate) were simulated. Next, PK of the three inhibitors was simulated when co-administered with strong CYP3A4 inhibitors, namely, ketoconazole, posaconazole, and itraconazole, as well as strong CYP3A4 inducer rifampicin. The final modeling parameters for midazolam and the four CYP3A4 modulators are provided in Supplementary Table S1, and the inhibition and induction parameters of the four modulators are listed in Supplementary Table S2. For the DDI simulations, the dosage regimens of the three ALK inhibitors, CYP3A4 inhibitors, and CYP3A4 inducer were designed based on the literature′s data (Xu et al., 2015b; Chen et al., 2020; Patel et al., 2020; Zhao et al., 2020) (see Table 4).

#### 2.2.3 Physiologically based pharmacokinetic model prediction evaluation

To assess the accuracy of predictions, the fold errors for AUC, C_max_, and C_trough_ were calculated by comparing the predicted values from the PBPK model with the corresponding observed values. Generally, a fold error ranging from 0.5 to 2.0 is considered indicative of accurate model predictions. If the fold error falls within this range, it suggests that the PBPK model provides reasonably accurate estimates.

### 2.3 Sensitivity analysis

The sensitivity analysis was performed to assess how selected model parameters influence the AUC, C_max_, and C_trough_. Patient received standard dose regimens of 250 mg BID for CRI, 600 mg BID for ALE, and 100 mg OD for LOR. The modeling parameters for the sensitivity analysis were chosen based on the following criteria: 1) optimized and 2) could have significant influence on the AUC, C_max_, and C_trough_. The selected parameters were 1) LogP, 2) f_up_’, 3)R_bp_’, 4) CL_int_ CYP3A4, 3A5, 2C8, 2C19, UGT1A3/1A4, 5) CYP3A4 V_max_ and CYP3A4 K_m_, 6) CL_int_ P-gp, 7) K_i_ CYP3A4, 8) EC_50_ and E_max_ for CYP3A4, and (9) expression (CYP3A4, CYP3A5, CYP2C8, CYP2C19, UGT1A3, UGT1A4, and P-gp).

The impacts of the selected parameters on the AUC, C_max_, and C_trough_ were evaluated by altering the value of each parameter by ± 20% (Saeheng et al., 2020). The sensitivity coefficient (SC) is computed as follows (Saeheng et al., 2020):

SC=∆Y/Y÷∆P/P, (8)

where ∆Y is the alteration of predicted AUC, C_max_, or C_trough_; Y is the initial value of predicted AUC, C_max_, or C_trough_; ∆P is the alteration of model parameters; and P is the initial value of assessed parameters. If a certain SC absolute value is above 1.0 (i.e., it means that a 20% change of the assessed parameters results in a 20% alteration in AUC, C_max_, or C_trough_), it means this model parameter has a significant influence on predicted AUC, C_max_, or C_trough_.

### 2.4 Plasma and intracranial ALK occupancy prediction

The ALK occupancy (AO) time profiles were calculated using the following Eq. 9 (Georgi et al., 2018):

$$\text{AO}=\frac{I_{free}}{\left(K_{i}+I_{free}\right)}\times 100,$$ (9)

where I_free_ (μM) represents the free drug concertation in the plasma or CSF. K_i_(μM) is the equilibrium dissociation constant. AO represents the percent level of ALK occupancy. In this work, AO in plasma and CSF by the three inhibitors on the wild-type and three most common mutations ALK were simulated. K_i_ values for the three inhibitors against ALK were approximated with reported IC_50_ values (Cui et al., 2011; Friboulet et al., 2014; Kodama et al., 2014; Song et al., 2015; Sabari et al., 2017; Chuang et al., 2019) at the K_m_-level of substrate ATP using the Cheng–Prusoff correction.

### 2.5 Virtual population demographic characteristics and dosing regimens

The demographic characteristics used in every simulation were that of the corresponding clinical study. The information of virtual population in PK-Sim includes age range, body weight, height, and proportion of female individuals. If the demographic characteristics, such as age range and gender proportions, are available from clinical studies, the actual data obtained from those studies would be used in the simulations. This approach ensures that the simulation aligns closely with the real-world characteristics of the subjects involved in clinical studies. If certain data were unavailable, PK-Sim uses common default values as surrogates. For example, age is set to a range of 30–70 years and the proportion of females is assumed to be 50%. In cases where the number of subjects in clinical studies is less than 10, 10 virtual subjects are created for the simulations to ensure a sufficient sample size. Table 2 provides information on the demographic characteristics of the virtual population, including age range and gender proportions. In addition, it lists the dosing regimens used in the simulations.

**TABLE 2:** Dosing regimens and demographic characteristics in the simulations of PBPK model development and validation.

Drug	Dosage schedules	Purpose	Virtual population	Number of virtual subjects^a^	Age range set (year)	Proportion of female (%)	Source of data or comments
CRI	50 mg intravenous dosing, SD	Validate plasma PK data when dosed alone	Healthy	14	18–55	0	Xu et al. (2015a)
	250 mg OD, SD						
	250 mg OD, RD for consecutive 14 days		Patients	10	40–75	78	Kurata et al. (2015)
	Multiple doses for 50–300 mg, RD for consecutive 28 days		Patients	10	25–73	42	Age range was set based on mean age of 49 (Clark et al., 2019)
	250 mg OD, SD		Healthy	10	26–53	0	Huiping et al. (2022)
	100 mg OD, RD for consecutive 14 days	Validate intracranial concentration	Patients	10	30–70	50	Demographic data were set in this study
	➀CRI: 150 mg, SD, on day 4	Validate plasma PK variation when dosed with CYP3A4 perpetrators	Healthy	16	26–53	0	Xu et al. (2015a)
	➁Ketoconazole: 200 mg BID, RD from days 1 to 16		Healthy	15	29–44	0	Xu et al. (2015b)
	➀CRI: 250 mg, SD, on day 9		Healthy	15	38–47	7	
	➁Rifampin: 600 mg OD, RD from days 1 to 14						
ALE	600 mg OD, SD	Validate plasma PK when dosed alone	Healthy	16	26–53	0	Morcos et al. (2017c)
	160, 240, and 300 mg BID, RD for consecutive 21 days		Patients	10	28–67	54	Seto et al. (2013)
	460, 600, and 900 mg BID, RD for consecutive 21 days		Patients	10	40–83	20	Gadgeel et al. (2014)
	600 mg OD, RD for days 1–14	Validate intracranial concentration	Patients	10	36–76	50	Age range was set based on mean age of 56 (Gainor et al., 2016)
	➀ALE: 300 mg, SD, on day 6	Validate plasma PK variation when dosed with CYP3A4 perpetrators	Healthy	10	30–70	50	Demographic data were set in this study
	➁Posaconazole: 400 mg BID, RD from days 1 to 14						
	➀ALE: 150 mg, SD, on day 6						
	➁Rifampin: 600 mg OD, RD from days 1 to 14						
LOR	50 mg intravenous dosing, SD	Validate plasma PK when dosed alone	Healthy	11	18–55	0	Hibma et al. (2022)
	100 mg OD, SD						
	100 mg OD, RD for consecutive 15 days		Patients	19	39–65	32	Chen et al. (2021)
	100 mg OD, SD			10	53–61	38	Lin et al. (2022)
	100 mg OD, RD for consecutive 21 days	Validate intracranial concentration	Patients	10	30–70	50	Sun et al. (2022)
	➀LOR: 150 mg, SD, on day 5	Validate plasma PK variation when dosed with CYP3A4 perpetrators	Healthy	16	20–54	0	Patel et al. (2020)
	➁Itraconazole: 200 mg BID, RD from days 1 to 11						
	➀LOR:150 mg, SD, on day 13		Healthy	12	21–55	8.3	Chen et al. (2020)
	➁Rifampin: 600 mg OD, RD from days 6 to 17						

BID, twice daily; SD, single dose; RD, repeated doses.

## 3 Results

### 3.1 Validation of the physiologically based pharmacokinetic models

#### 3.1.1 Validation using PK profiles and data

**FIGURE 2:**
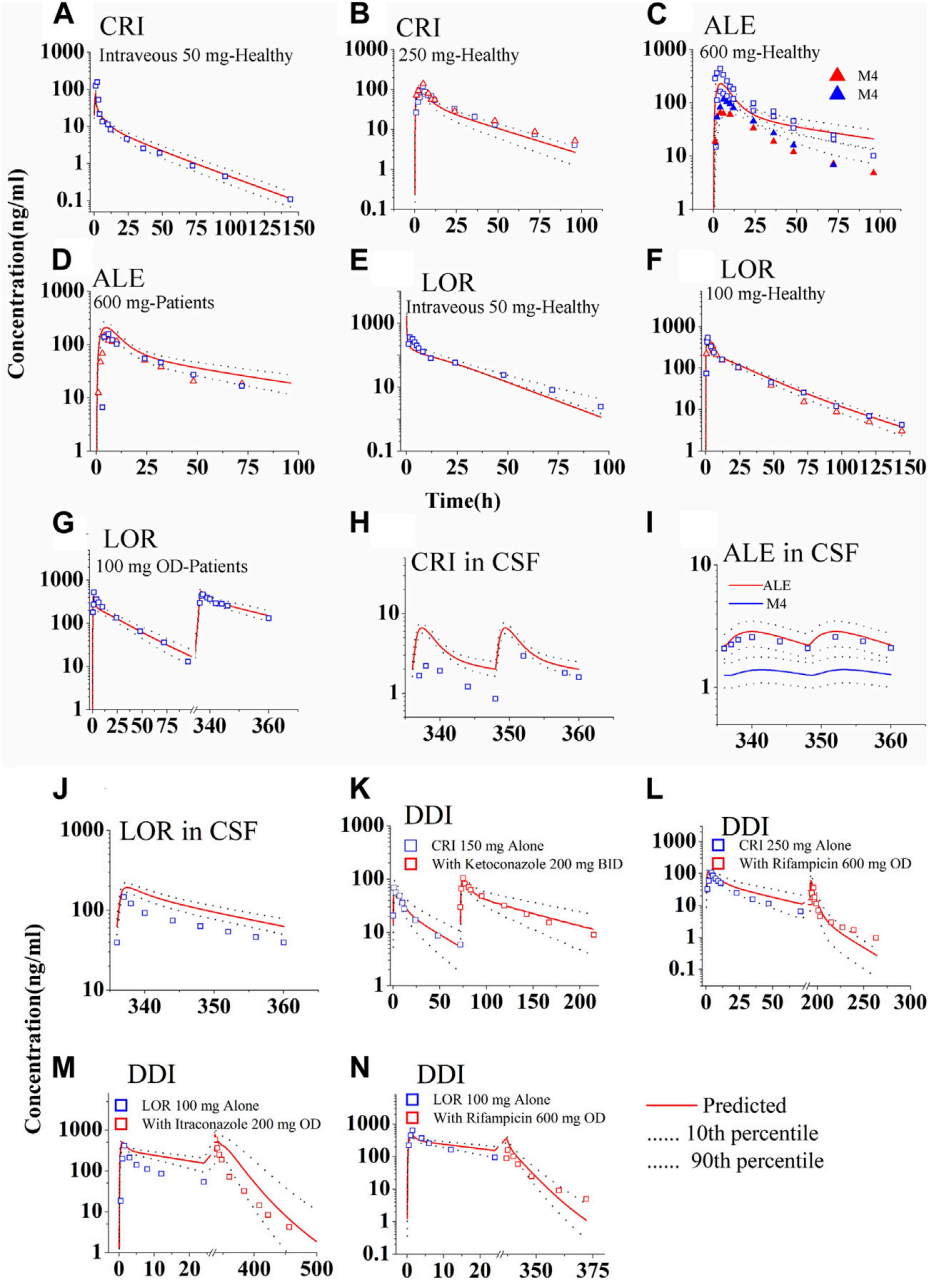
Simulations of the pharmacokinetics of the three ALK inhibitors after administration of single dose or repeated doses. The blue and red squares (parent drug) and solid up-triangles (metabolite M4) are the clinically observed data. The observed data in the CSF were derived by multiplying the plasma PK data with the observed K_CSF,P_ values. Panel **(A, B)** for CRI; Panel **(C, D)** for ALE; Panel **(E–G)** for LOR; Panel **(H–J)** for concentration in CSF of the three ALK inhibitors, respectively; Panel **(K, L)** for DDIs of CRI with ketoconazole and rifampicin; Panel **(M, N)** for DDIs of LOR with Itraconazole and rifampicin.

Figure 2 shows the predicted and observed plasma concentration–time profiles for CRI following intravenous and oral administration in healthy subjects (Figures 2A,B), ALE following oral administration in healthy subjects and NSCLC patients (Figures 2C,D), and LOR following intravenous and oral administration in healthy subjects and NSCLC patients (Figures 2E–G). The simulations demonstrated that the PBPK models for both healthy and diseased states were able to replicate the observed PK profiles (Gadgeel et al., 2014; Xu et al., 2015a; Morcos et al., 2017a; Morcos et al., 2017b; Morcos et al., 2017c; Clark et al., 2019; Stypinski et al., 2020; Chen et al., 2021; Hibma et al., 2022; Huiping et al., 2022; Lin et al., 2022). In Table 3, it can be observed that all ratios of AUC, C_max_, and C_trough_ fell within the range of 0.5–2.0.

**TABLE 3:** Comparisons of the geometric mean plasma and intracranial PK parameters between predicted and observed data in healthy and ALK-positive NSCLC patients.

PK	Clinical study	Drug	Dosing regimens	N	Subjects	AUC (ng·h/mL, CV%/±SD^a^)		C_max_ (ng/mL, CV %/±SD)		C_trough_ (ng/mL, CV %/±SD)		Prediction/observation ratio		
						Prediction	Observation	Prediction	Observation	Prediction	Observation	AUC	C_max_	C_trough_
Plasma	Xu et al. (2014)	CRI^b^	250 mg OD	14	Healthy	1968 (46)	2321 (34)	115 (43)	99.6 (28)	-	-	0.85	1.15	-
	Kurata et al. (2015)		500 mg OD	9	Patients	8738 (58)	-	849 (49)	-	630 (427–789)	508.5 (243.5–847.8)	-	-	1.24
	Clark et al. (2019)		50 mg OD	3	Patients	287 (48)	206 (64)	31 (42)	24 (52)	9 (5–14)	8 (5–11)	1.39	1.29	1.13
			100 mg OD	4		987 (43)	1087 (37)	71 (47)	86 (69)	26 (14–36)	31 (24–52)	0.91	0.83	0.84
			200 mg OD	8		2473 (51)	2047 (48)	165 (48)	149 (27)	69 (42–93)	44 (31–160)	1.21	1.11	1.57
			200 mg BID	4		2915 (51)	1780 (61)	290 (46)	189 (48)	205 (134–263)	158 (132–183)	1.64	1.53	1.30
			250 mg BID	4		3820 (45)	3084 (32)	378 (46)	327 (25)	271 (178–344)	259 (159–356)	1.24	1.16	1.05
			300 mg BID	5		4421 (50)	4067 (55)	440 (46)	420 (48)	311 (215–396)	279 (183–403)	1.09	1.05	1.11
	Xu et al. (2022)		250 mg OD	8	Healthy	2239 (38)	2438 (42)	126 (36)	118.5 (31)	-	-	0.92	1.06	-
	Seto et al. (2013)	ALE^c^	160 mg BID	3	Patients	2029 ± 736	2310 ±598	187 ± 69	300 ± 104	145 ± 52	214 ± 34	0.88	0.62	0.68
			240 mg BID	3		3179 ± 1159	2970 ± 937	291 ± 107	385 ± 100	230 ± 82	262 ± 115	1.07	0.76	0.88
			300 mg BID	6		4081 ± 1491	4970 ± 3260	374 ± 137	575 ± 322	296 ± 106	463 ± 369	0.82	0.65	0.64
	Gadgeel et al. (2014)		460 mg BID	7		6629 ± 2427	4980 ± 1340	604 ± 223	618 ± 165	486 ± 174	460 ± 130	1.33	0.98	1.06
			600 mg BID	5		9013 ± 3300	5400 ± 1400	819 ± 302	676 ± 186	665 ± 238	502 ± 142	1.67	1.21	1.32
			900 mg BID	7		11901 ± 4355	9840 ± 4620	1078 ± 397	1140 ± 448	883 ± 315	822 ± 444	1.21	0.95	1.07
	Morcos et al. (2016)		600 mg OD	6	Healthy	5916 (31)	6090 (13)	223 (25)	175 (11)	-	-	0.97	1.27	-
	Morcos et al. (2017)			48		5757 ± 874	4360 ± 1160	215 ± 55	204 ± 57	-	-	1.32	1.05	-
	Morcos et al. (2018)			24		5213 ± 698	3180 ± 876	193 ± 46	169 ± 47	-	-	1.64	1.14	-
		M4	600 mg BID	48	Healthy	2205 ± 796	1890 ± 477	80 ± 23	65 ± 17	-	-	1.17	1.23	-
				24		2756 ± 994	3480 ± 758	85 ± 31	126 ± 32	-	-	0.79	0.67	-
	Shaw et al. (2017)	LOR^b^	10 mg OD	2	Patients	913 (42)	820^d^	69 (44)	74^d^	26 (21)	-	1.11	0.93	-
			25 mg OD	3		2547 (41)	1708 (29)	156 (31)	138 (35)	53 (39)	-	1.49	1.13	-
			50 mg OD	3		3479 (36)	3487 (41)	293 (23)	360 (27)	88 (22)	-	1.00	0.81	-
			75 mg OD	11		4894 (42)	4117 (55)	337 (21)	422 (50)	119 (28)	-	1.19	0.80	-
			100 mg OD	14		5611 (48)	5065 (32)	559 (28)	569 (32)	150 (55)	-	1.11	0.98	-
			150 mg OD	2		6515 (41)	6185^d^	825 (27)	638^d^	206 (46)	-	1.05	1.29	-
			200 mg OD	2		12011 (58)	7856^d^	1101 (32)	1042^d^	272 (32)	-	1.53	1.06	-
	Stypinski et al. (2020)		100 mg OD	6	Healthy	9166 (41)	7600 (26)	526	600 (18)	-	-	1.21	0.88	-
	Hibma et al. (2022)			11		8632 (36)	8289 (34)	442	501 (38)	-	-	1.04	0.88	1.58
	Chen et al. (2021)			19	Patients	7819 (30)	9088 (35)	581 (31)	695 (40)	158 (31)	100 (32)	0.86	0.84	-
	Lin et al. (2022)			8	Patients	7055 (35)	8329 (33)	563 (29)	547 (48)	152 (28)	-	1.11	0.93	-
CSF	Costa et al. (2011)	CRI	250 mg BID	1	patients	17.0 ± 2.2	-	6.7 ± 0.90	-	2.0 ± 0.52	0.62	-	-	3.23
	Metro et al. (2016)	ALE	600 mg BID	2	patients	31.2 ± 6.0	-	2.9 ± 0.58	-	2.2 ± 0.28	1.40	-	-	1.57
	Sun, et al. (2022)	LOR	100 mg OD	4	patients	2784 ± 360	-	218 ± 22.3	-	63.0 ± 11.1	86.5 ± 36.8	-	-	0.73

^a^ CV %, percentage coefficient of variation; SD, standard deviation.

^b^ Geometric mean values are shown.

^c^ Arithmetic mean values are shown.

^d^ Not reported data.

The predicted and calculated free concentration–time profiles in CSF are shown in Figures 2H–J. The simulations indicated a slight overestimation of CSF concentrations for CRI and LOR (Figures 2H,J), whereas the 90% prediction interval (CI) of the population PBPK model could cover variations observed for ALE (Figure 2I). Notably, the plasma exposure and C_max_ of LOR were considerably higher than those of CRI and ALE, as shown in Figures 2H–J. Furthermore, the CSF concentration of ALE closely resembled that of CRI. Analyzing the data presented in Table 3, it is evident that the C_trough_ ratio in CSF for CRI exceeds 2.0, whereas the C_trough_ ratios of ALE and LOR in CSF fall within the range of 0.5–2.0. Overall, these simulation results align with the clinical observations, specifically for CRI (predicted 2.0 vs. observed 0.62 ng/mL (Costa et al., 2011)), ALE (predicted 2.2 vs. observed 1.4 ng/mL (Metro et al., 2016)), and LOR (predicted 63.0 vs. mean observed 86.5 ng/mL (Sun et al., 2022))。

#### 3.1.2 Verification using drug–drug interaction simulations

Supplementary Figure S1 and Supplementary Table S3, respectively, present the predicted and clinically observed PK profiles and data for midazolam and four CYP3A4 modulators. The DDI simulations of CRI and LOR are shown in Figures 2K–N. With the exception of the PK of LOR co-administered with itraconazole, which exhibited greater variability, other simulations demonstrated that the observed data fell within the 90% CI of the population PBPK model-predicted levels. The ratios predicted by the PBPK models are summarized in Table 4. Except for the C_max_ ratio (0.54 vs. 0.24) of LOR co-administered with rifampin, the other predicted AUC_0-inf_ and C_max_ ratios are in good agreement with the clinically observed ratios (Xu et al., 2015b; Chen et al., 2020; Patel et al., 2020; Zhao et al., 2020). These DDI simulations further confirmed that the CYP3A4 metabolic parameters of the three inhibitors are appropriately incorporated into the PBPK model. Moreover, Supplementary Table S4 provides the predicted AUC and C_max_ ratios of midazolam when co-administered with CRI and LOR, respectively. The good consistency observed between the predicted and observed ratios indicates that the inhibition and induction parameters of CRI and LOR on CYP3A4 are appropriate in the PBPK model.

**TABLE 4:** PK changes (geometric mean, CV%) of crizotinib, alectinib, and lorlatinib under DDIs.

Clinical study	Parameters	CRI only (150 mg OD)	CRI + ketoconazole (+200 mg BID)	Predicted ratio	Observed ratio
Xu et al. (2015b)	AUC_0-inf_ (ng·h·mL^-1^)	1590 (33)	5452 (43)	3.43	3.16
	C_max_ (ng/mL)	70 (28)	100 (33)	1.43	1.44
	Parameters	CRI only (250 mg OD)	CRI + rifampin (+600 mg OD)	Predicted ratio	Observed ratio
	AUC_0-inf_ (ng·h·mL^-1^)	2943 (30)	388 (33)	0.13	0.18
	C_max_ (ng/mL)	121 (19)	60 (33)	0.50	0.31
Zhao et al. (2020)	Parameters	ALE only (300 mg OD)	ALE + posaconazole (+400 mg BID)	Predicted ratio	Observed ratio
	AUC_0-inf_ (ng·h·mL^-1^)	3280 (32)	7007 (49)	2.14	1.75
	C_max_ (ng/mL)	136 (44)	182 (34)	1.34	1.18
	Parameters	ALE only (600 mg OD)	ALE + rifampin (+600 mg OD)	Predicted ratio	Observed ratio
	AUC_0-inf_ (ng·h·mL^-1^)	5146 (45)	999 (47)	0.19	0.27
	C_max_ (ng/mL)	260 (28)	116 (31)	0.45	0.49
	Parameters	LOR only (100 mg OD)	LOR + itraconazole (+200 mg OD)	Predicted ratio	Observed ratio
Patel et al. (2020)	AUC_0-inf_ (ng·h·mL^-1^)	5580 (21)	8287 (39)	1.49	1.41
	C_max_ (ng/mL)	455 (18)	649 (37)	1.43	1.24
	Parameters	LOR only (100 mg OD)	LOR + rifampin (+600 mg OD)	Predicted ratio	Observed ratio
Chen et al. (2020)	AUC_0-inf_ (ng·h·mL^-1^)	7021 (23)	1726 (58)	0.25	0.15
	C_max_ (ng/mL)	692 (32)	374 (29)	0.54	0.24

In summary, the DDI simulations demonstrated that the population PBPK models are able to accurately predict the AUC, C_max_, and plasma/intracranial C_trough_ in healthy and diseased population.

### 3.2 Sensitivity analysis

**FIGURE 3:**
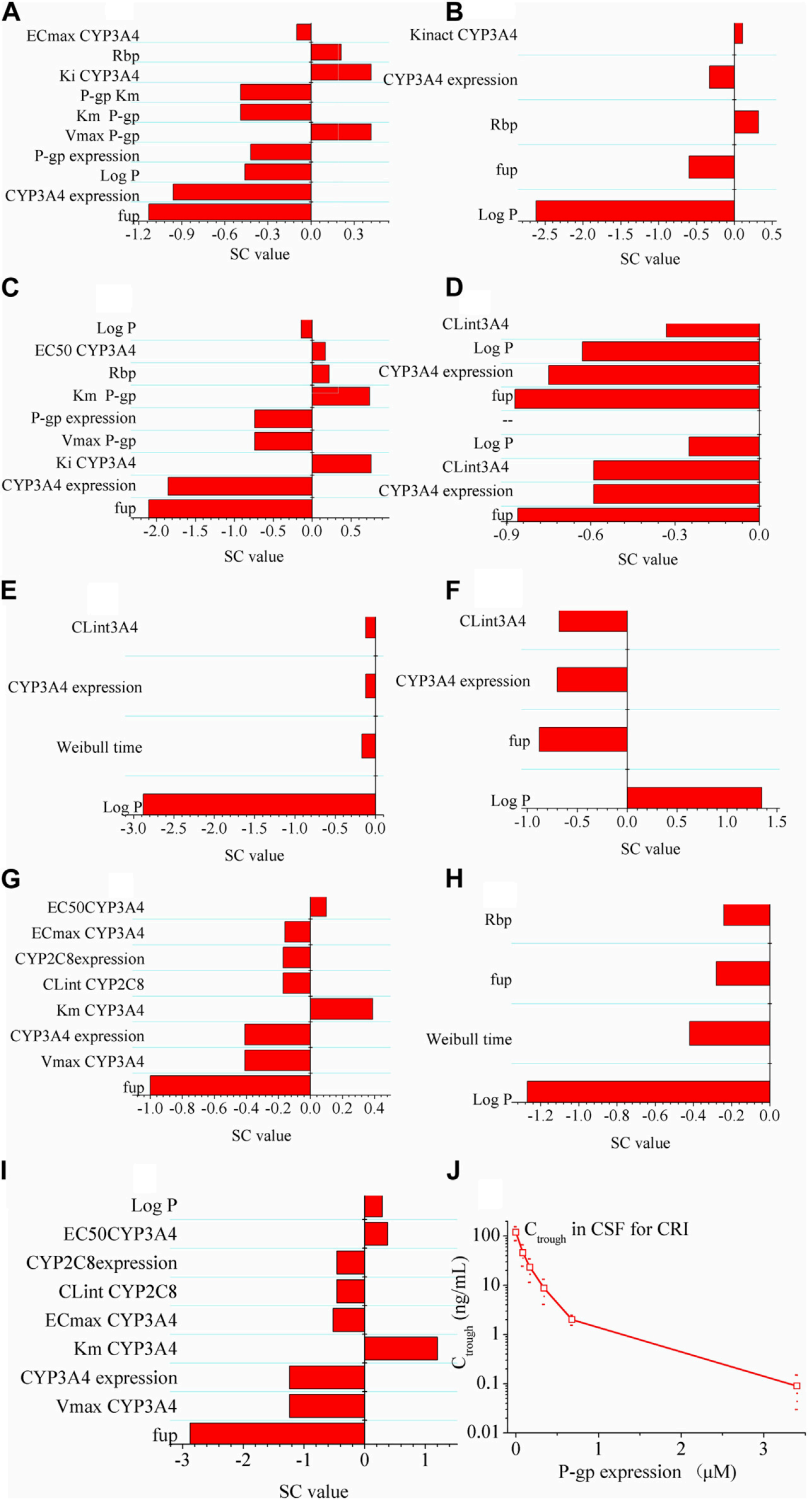
Sensitivity analysis of the diseased PBPK models. Panel **(A–C)** show effect of modeling parameters of CRI on AUC, C_max_ and C_trough_ of CRI; Panel **(D–F)** show effect of modeling parameters of ALE on AUC, C_max_ and C_trough_ of ALE; Panel **(G–I)** show effect of modeling parameters of LOR on AUC, C_max_ and C_trough_ of LOR. If the absolute value of the SC is greater than 1.0, it indicates that the corresponding model parameter has a significant influence on the AUC, C_max_, or C_trough_. Panel **(J)** illustrates the effect of P-gp on the intracranial Ctrough (minimum concentration) of CRI (the drug). It indicates that as P-gp expression increases, the Ctrough of CRI in cerebrospinal fluid (CSF) decreases.

As shown in Figure 3, log P exhibited the highest sensitivity as a parameter for predicting C_max_ of the ALK inhibitors. For CRI, the most sensitive parameter affecting AUC was f_up_. However, no specific sensitive parameters were identified for AUC of ALE and LOR. In terms of C_trough_ of CRI in NSCLC patients, f_up_ and CYP3A4 expression were the most sensitive parameter. Log P was found to have the greatest impact on C_trough_ of ALE. As for C_trough_ of LOR, the most sensitive parameters were f_up_, V_max_ CYP3A4, CYP3A4 expression, and K_m_ CYP3A4. The sensitivity analysis conducted on both the healthy and diseased PBPK models indicated similar results, with the exception of CYP3A4 expression which did not exhibit sensitivity to the C_trough_ of CRI in healthy subjects (information not provided in this study).

Next, the simulations conducted to assess the influence of P-gp expression on the intracranial C_trough_ of CRI revealed notable findings. Figure 3J illustrates the influence of P-gp expression within the range of 0–3.4 μM on the C_trough_ in CSF. The results clearly demonstrate that the P-gp expression at the blood–brain barrier significantly affects the intracranial C_trough_ of CRI. As the P-gp expression increases, the intracranial C_trough_ gradually decreases. This effect is evident with a substantial 60-fold increase when P-gp efflux is absent at the blood–brain barrier.

### 3.3 Plasma and intracranial ALK occupancy prediction

**FIGURE 4:**
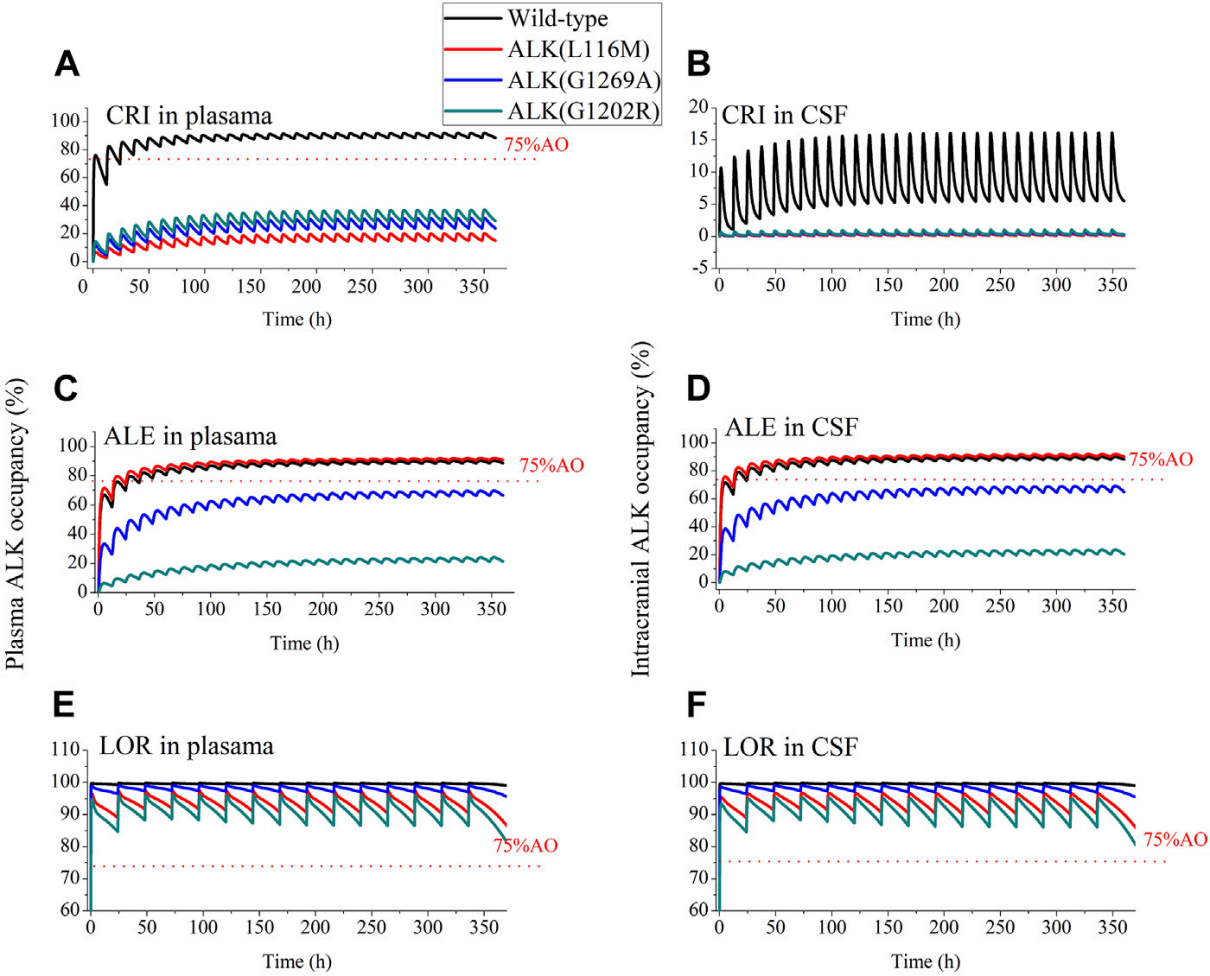
Simulations of wild-type and three mutations ALK occupancy in plasma and CSF by crizotinib, alectinib and lorlatinib. The plasma and intracranial ALK occupancy of CRI [Panel **(A, B)**, 250 mg BID], ALE [Panel **(C, D)**, 600 mg BID], and LOR [Panel **(E, F)**, 100 mg OD].

According to the study by Yamazaki (2013), >75% ALK inhibition was required in NSCLC patients for CRI to produce clinically higher ORR. As a result, >75% AO was defined as an effective threshold for the three ALK inhibitors in this work. Figure 4 shows the AO time course in both plasma and CSF following 14 consecutive days of dosing with the inhibitors. For CRI, only plasma AO is greater than 75% in patients with wild-type ALK (Figures 4A,B). The ALK engagement by CRI in plasma was markedly higher than ALK engagement by CRI in CSF. In contrast, the ALK engagements by ALE and LOR were independent of plasma and CSF, likely due to their high penetration into CSF. Notably, ALE achieved more than 75% ALK occupancy in both plasma and CSF for wild-type ALK and mutation ALK^L1196M^. This simulation is consistent with the clinical trial in which ALE demonstrated efficacy in NSCLC patients with brain metastases (Jessica et al., 2019). The simulation suggests that ALK^G1202R^ was most likely to confer resistance to ALE, as the maximal AO was less than 20% (Figures 4C,D). On the other hand, LOR showed more than 75% occupancy of wild-type ALK and three mutations (Figures 4E,F). The study indicates that LOR can overcome resistance to the first- and second-generation ALK inhibitors, even in cases mediated by ALK^G1202R^, and may have significant activity on brain metastasis. The AO simulations of LOR were also in agreement with the clinical study (Food and Drug Administration FDA, 2011c).

### 3.4 Simulations of appropriated dosing regimens for the three inhibitors

**FIGURE 5:**
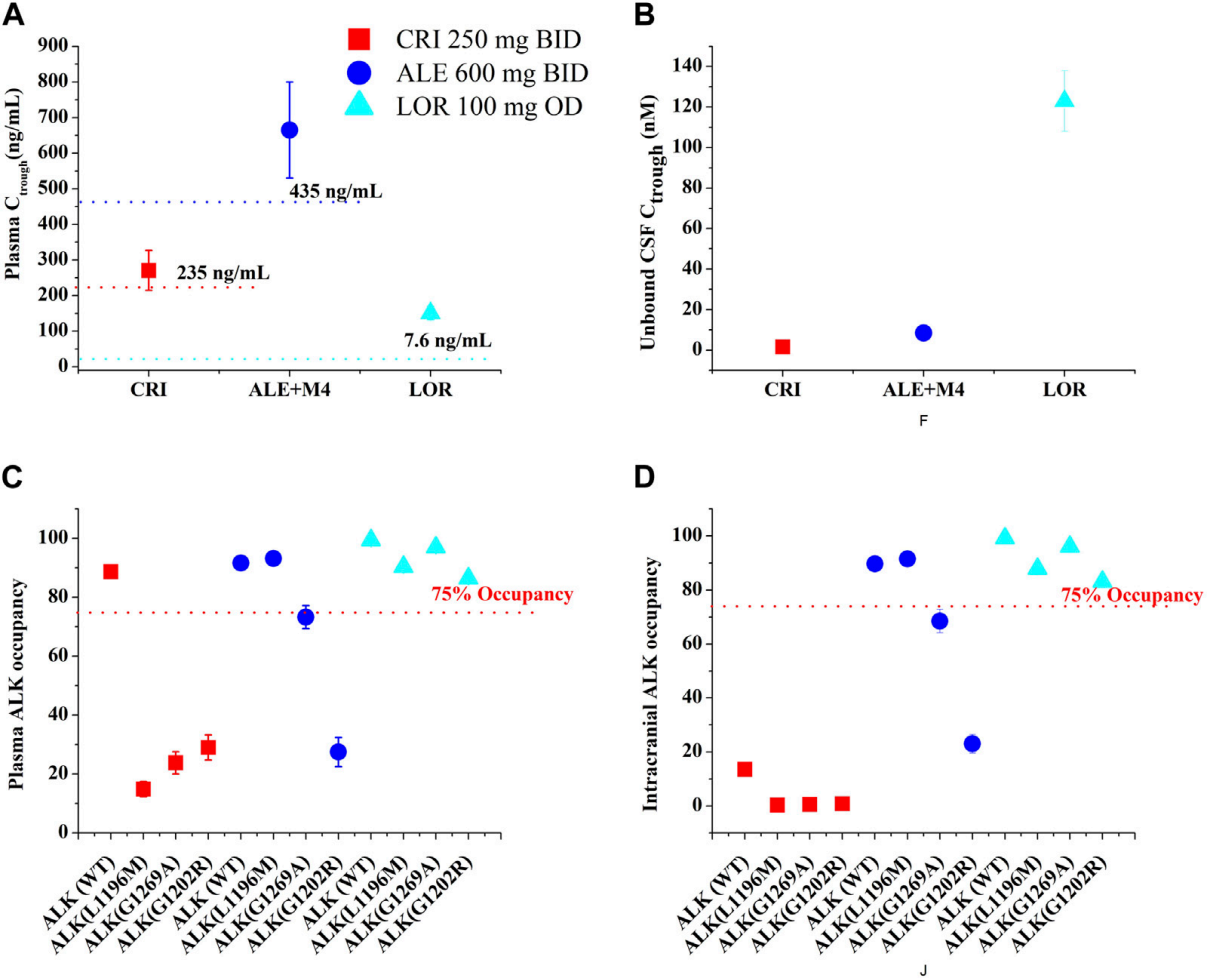
The PBPK simulations of predicted C_trough_ [plasma, **(A)**; CSF, **(B)**] and ALK occupancy [plasma, **(C)**; intracranial, **(D)**] in NSCLC patients. Data were shown as geometric mean values and 95% CI.

The exposure–response relationships for efficacy in patients have suggested that the clinical efficacy of three inhibitors is strongly correlated with their steady-state C_trough_. Minimum C_trough_ of ≥235 ng/mL was obtained for CRI (Groenland et al., 2021), ≥435 ng/mL for ALE (Groenland et al., 2021), and 7.6 (wild-type)/62 (ALK^L1196M^)/150 (ALK^G1202R^) ng/mL for LOR (Shaw et al., 2017) as a PK threshold for optimal clinical efficacy. Figure 5 and Table 5 illustrate C_trough_ and AO in both plasma and CSF based on the clinically proposed dosing regimens for the three inhibitors. For CRI, the simulations suggested that a dose of 250 mg BID is appropriate for inhibiting wild-type ALK. However, higher doses of CRI could also not achieve clinical efficacy against the three ALK mutations. Similarly, the simulations support the appropriateness of the proposed dose of 250 mg BID for ALE. In the case of ALK^G1269A^ mutation, increasing the dose may be a more effective option for clinical therapy. Furthermore, the simulations demonstrated that the proposed dosing regimens of 100 mg OD are appropriate for NSCLC patients with wild-type ALK and three ALK mutations, even in the presence of brain metastasis. These findings provide additional evidence supporting the clinical efficacy of the recommended dosing regimens for the three inhibitors in various patient populations.

**TABLE 5:** Mean trough concentration unbound and ALK occupancy at steady state in plasma and CSF in ALK-positive NSCLC patients.

Drug	Dosing regimen	Therapeutic target	ALK occupancy threshold (%)	C_etv_ (ng/mL)	K_i_(nM) against ALK	Unbound C_trough_ (nM, 90% CI)		Minimal ALK occupancy (%, 90% CI)	
						Plasma	Unbound CSF	Plasma	Intracranial
CRI	250 mg BID	ALK (wild-type)	75	235	10	78.2 (61.9, 94.5)	1.56 (1.24, 1.89)	88.7 (86.1, 90.4)	13.5 (11.0, 15.9)
		ALK (L1196M)		-	446			14.9 (12.2, 17.5)	0.3 (0.28, 0.42)
		ALK (G1269A)		-	250			23.8 (19.8, 27.4)	0.6 (0.49, 0.75)
		ALK (G1202R)		-	191			29.0 (24.5, 33.1)	0.8 (0.65, 0.98)
ALE + M4	600 mg BID	ALK (WT)		435	1.0	11.0 (8.8, 13.1)	8.5 (6.8, 10.1)	91.7 (89.8, 94.2)	89.7 (87.2, 91.0)
		ALK (L1196M)		-	0.8			93.2 (91.7, 80.9)	91.6 (89.5, 92.7)
		ALK (G1269A)		-	4.0			73.3 (68.8, 76.6)	68.5 (63.0, 71.6)
		ALK (G1202R)		-	29			27.5 (23.3, 31.1)	23.0 (19.0, 25.8)
LOR	100 mg OD	ALK (WT)		7.6	1.0	160 (140, 179)	123 (108, 138)	99.5 (99.5, 99.7)	99.4 (99.3, 99.5)
		ALK (L1196M)		62	17			93.8 (93.0, 94.5)	92.1 (91.1, 92.9)
		ALK (G1269A)		-	5.0			94.9 (94.3, 95.5)	93.5 (92.7, 94.2)
		ALK (G1202R)		150	25			83.1 (81.2, 84.6)	79.1 (76.9, 80.9)

-, not reported data; C_etv_, effective PK threshold values.

## 4 Discussion

In this study, the effective thresholds of plasma C_trough_ (CRI: 235 ng/mL, ALE: 435 ng/mL, and LOR: 7.6, 622, 150 ng/mL in wild-type, ALK^L1196M^, and ALK ^G1202R^ mutations, respectively) and AO (>75%) in both plasma and CSF were defined. The developed PBPK models were able to accurately predict the plasma and intracranial C_trough_ for the three inhibitors in healthy individuals and in NSCLC patients. The simulations have been demonstrated by multiple clinical PK study data (see Figure 2; Table 3). To our knowledge, this is the first study to assess the PK and AO of the three ALK inhibitors in the plasma and CSF of NSCLC patients. In cancer patients, known physiological differences in CYP enzyme expression, plasma protein level, and hematocrit have been reported (Dixon et al., 2003; Schwenger et al., 2018). In addition, reductions in the plasma protein level and hematocrit in patients can result in increased f_up_ and R_bp_ (see Table 1), which were modified in the mode for patients. Finally, the five modeling parameters (CYP3A4 and CYP2C19 expression, plasma protein level, hematocrit, f_up_, and R_bp_) were incorporated into the diseased PBPK model, whereas the remaining parameters were assumed to be identical to the healthy condition.

The sensitivity analysis conducted in this study identified f_up_ and CYP3A4 expression as sensitive parameters for the three ALK inhibitors in most cases. Therefore, it was necessary to modify the f_up_ and CYP3A4 expression values in the diseased PBPK model to accurately represent the effects of these parameters in patients. In addition, the simulations demonstrated that P-gp expression at the blood–brain barrier plays a significant role in determining C_trough_ of CRI in the CSF (Figure 3J). This finding suggests that low penetration of CRI into the brain is primarily attributed to the presence of P-gp, which limits its distribution across the blood–brain barrier. Overall, these results indicate the importance of considering factors such as f_up_, CYP3A4 expression, and P-gp expression when modeling the PK and distribution of the three ALK inhibitors, particularly regarding their penetration into the CSF.

It is noteworthy that CRI and ALE can inhibit their own metabolism through time-dependent inhibition of CYP3A4 as well as increase their own metabolism through induction of *in vivo* CYP3A4 expression (interaction parameters in Table 1). On the other hand, LOR can only enhance its own metabolism through auto-induction of CYP3A4 expression (interaction parameters in Table 1), with low auto-inhibition of CYP3A4. In this work, the PBPK models incorporated CYP3A4 auto-inhibition (K_i,_ k_inact_) and auto-induction parameters (E_max_ and EC_50_) to ensure the predictive performance of the model. However, this may not be robust in this case. Recent studies (Yamazaki et al., 2015; Hanke et al., 2018) have also applied this approach to predict the clinical PK for mixed CYP3A4 inhibitors and inducers, further supporting the need to incorporate these mixed inhibition and induction parameters into our developed PBPK models. The PBPK models predicted that mean oral clearance (CL) following a single oral 100 mg dose increased by 1.78-fold due to auto-induction compared with the CL at the steady state, which is in agreement with the clinical data (1.78-fold vs. 1.64-fold) (Food and Drug Administration FDA, 2011c). These results suggest that it is necessary to consider the complex interplay of auto-inhibition and auto-induction when developing PBPK models.

Approximately 30% of ALK-positive patients with NSCLC are likely to develop brain metastases (Zou et al., 2022). Unbound drug concentration in the CSF is often used as a surrogate for concentration at the target in clinical setting (De Lange and Danhof, 2002). The predictive power of concentration of the three inhibitors in the CSF has been demonstrated by our developed PBPK models. According to the PBPK model, the mean C_trough_ (8.5 nM) for ALE and C_trough_ (123 nM) for LOR in the CSF were higher than the concentration required for three (ALE) and four (LOR) ALK inhibition (see Table 5). In contrast, the PBPK model showed that the mean C_trough_ for CRI in CSF (1.56 nM) was lower than the concentration required for wild-type and four mutations of ALK inhibition (see Table 5).

Multiple molecular mechanisms can lead to resistance to the first- and second-generation ALK inhibitors (Recondo et al., 2020). Among these, approximately 50% of resistance cases are attributed to on-target resistance, specifically ALK resistance mutations (Yoda et al., 2018). The two key factors conferring on-target resistance are K_i_ against the ALK mutation and unbound concentration on target cells. In this study, we calculated AO using K_i_ plus unbound concentration in CSF, which can help explain the mechanism of on-target resistance. A few studies have been conducted to define a common value of at least level target occupancy for minimal efficacy target engagement, such as at least 90% occupancy for soluble epoxide hydrolase (Lee et al., 2019), more than 90% occupancy for BTK (Food and Drug Administration FDA, 2011d), and >70% occupancy for α-glucosidase (Wang et al., 2019). In our study, we defined >75% AO for reaching the optimal therapeutic level for ALK inhibitors. The low AO against three mutations in plasma and four ALK mutations in CSF explains the resistance of CRI to ALK mutations and brain metastasis. In addition, the PBPK model of CRI demonstrated that P-gp efflux at the blood–brain barrier restricts its accumulation in the brain. This simulation agrees with the previous work (K_CSF,P_ increased by 13.9-fold after P-gp was knocked out) (Tang et al., 2014). Furthermore, the model predicted ALE resistance to ALKG^1202R^ (see Table 5; Figure 5), which is also in agreement with the study by Ou et al. (2014).

The appropriate dosing regimens for NSCLC populations were investigated for the three inhibitors based on the geometric mean and 95% CI of predicted C_trough_ and AO (see Figure 5). This strategy for optimal dosing has been proposed by Adiwidjaja et al., 2022. Based on this strategy, it was suggested that CRI 250 mg BID, ALE 600 mg BID, and LOR 100 mg OD in NSCLC patients could represent the optimal dosing regimens (see Figure 5). In cases where patients have the ALKG_1269A_ mutation, increasing the dose of ALE may lead to a better clinical ORR. Furthermore, when administered concurrently with CYP3A4 inhibitors and inducers, the PBPK models can also provide the appropriate dosage regimens for the three inhibitors.

There are still some limitations to the present model. First, the PBPK models used for diseased conditions do not consider the role of other physiological parameters, except for the five modeling parameters mentioned earlier. Second, the effect of ALK overexpression in patients on AO has not been evaluated yet. In mice, ALK expression has been shown to play an important role in ALK inhibition in the brain (Shaw et al., 2017), but its effect in human patients is uncertain. Third, there is uncertainty due to incomplete equivalence between the free concentration in interstitial fluid and that in CSF, which is important to be recognized when using the PBPK model for simulations.

## 5 Conclusion

In summary, we have successfully developed both healthy and diseased PBPK models for CRI, ALE, and LOR. These models adequately predicted the concentration and AO of the three ALK inhibitors in the plasma and CSF of NSCLC patients. In addition, PBPK models enable us to analyze the mechanisms of on-target resistance and determine appropriate dosing regimens for these inhibitors.

## Data Availability

The original contributions presented in the study are included in the article/Supplementary Material; further inquiries can be directed to the corresponding authors.
