# Supplementary material for: Prediction of trough concentration and ALK occupancy in plasma and cerebrospinal fluid using physiologically based pharmacokinetic modeling of crizotinib, alectinib, and lorlatinib
Source: Front Pharmacol. 2023 Nov 22;14:1234262. doi: 10.3389/fphar.2023.1234262 (PMC10703149; doi:10.3389/fphar.2023.1234262)
Supplement: Supplementary file 1 [file Table1.docx]

**Supplementary Table S1** Inputting parameters used for the PBPK models of substrate and modulators in DDI simulations

| Property | Values | | | | | | | |
| --- | --- | --- | --- | --- | --- | --- | --- | --- |
|  | Midazolam^a^ | Ketoconazole^a^ | | Itraconazole/Hydroxy-itraconazole^b^ | Rifampicin^a^ | Posaconazole^c^ |  |  |
| MW(g·mol^-1^) | 325.8 | 531.4 | | 705.6/721.7 | 822.94 | 700.79 |  |  |
| pKa | 10.9(Acid), 6.2(Base) | 6.51(base) | | 3.7/2.53,4.91(base) | 1.7(acid), 7.9(base) | 2.9/4.1(base) |  |  |
| Log P | 2.90 | 2.67(@pH7.4) | | 4.2/3.5 | 2.3 | 4.0 |  |  |
| Solubility(μg/mL) | 50 (@pH6.5) | 6.93(@pH6.5) | | 5.4(@pH1.2)/- | 2.8(@pH7.5) |  |  |  |
| P_eff_ (🞨10^-5^ cm⋅s^-1^) | 1.55 | 1.24 | | - | 1.24 | 0.038 |  |  |
| P_app_ (🞨10^-6^ cm⋅s^-1^) | - | - | | 57.1/- | - | - |  |  |
| f_up_ | 0.03 | 0.015 | | 0.016/0.021 | 0.17 | 0.01 |  |  |
| Rbp | 0.68 | 0.59 | | 0.58/0.58 | 0.89 | 0.62 |  |  |
| Intravenous CL (L/h) | - | - | | - | - | 7.32 |  |  |
| CYP3A4 CL_int_ (pmol/min/mg protein) | 550.0 | - | | - | - | - |  |  |
| CYP3A4 CL_int_ (μl/min/pmol) | - | - | | - | - | - |  |  |
| UGT2B7 CL_int_（L/min） | - | - | | - | - | - |  |  |
| CYP3A4 V_max_ (pmol/min/pmol) | - | 8.0 | | 0.65/0.05 | - | - |  |  |
| CYP3A4 K_m_ (μM) | 4.0 | 15.0 | | 0.039/0.027 | - | - |  |  |
| CYP3A5 V_max_ (pmol/min/pmol) | - | - | | - | - | - |  |  |
| CYP3A5 K_m_ (μM) | - | - | | - | - | - |  |  |
| CYP1A2 V_max_ (pmol/min/pmol) | - | - | | - | - | - |  |  |
| CYP1A2 K_m_ (μM) | - | - | | - | - | - |  |  |
| CYP2A6 V_max_ (pmol/min/pmol) | - | - | | - | - | - |  |  |
| CYP2A6K_m_ (μM) | - | - | | - | - | - |  |  |
| CYP2B6 V_max_ (pmol/min/pmol) | - | - | | - | - | - |  |  |
| CYP2B6K_m_ (μM) | - | - | | - | - | - |  |  |
| CYP2C8 CL_int_ (μl/min/pmol） | - | - | | - | - | - |  |  |
| CYP2C9 CL_int_(μl/min/pmol） |  | - | | - | - | - |  |  |
| UGT1A1 V_max_ (pmol/min/pmol) | - | 9.37 | | - | - |  |  |  |
| UGT1A1 K_m_ (μM) | - | 22.3 | | - | - | - |  |  |
| UGT1A4 V_max_ (pmol/min/mg protein) | 276.0 | - | | - | - | - |  |  |
| UGT1A4 K_m_ (μM) | 37.8 | - | | - | - | - |  |  |
| AADAC V_max_ (μM/min) | - | - | | - | 9.87 | - |  |  |
| AADAC K_m_ (μM) | - | - | | - | 195.10 | - |  |  |
| P-gp V_max_ (μM/min) | - | - | | - | 0.036 | - |  |  |
| P-gp K_m_ (μM) | - | - | | - | 55.0 | - |  |  |
| OATP1B1 V_max_ (μM/min) | - | - | | - | 0.086 | - |  |  |
| OATP1B1K_m_ (μM) | - | - | | - | 1.5 | - |  |  |
| CL_R_(L/h) | 0.64 | GFR*f_u_ | | | |  |  |  |
| GFR fraction | - | 1.0 | | 1.0 | 1.0 | 1.0 |  |  |
| K_p_ scale | 3.0 | - | | 0.5/2.0 | 5.0 | - |  |  |
| Partition coefficients | Rodgers and Rowland | | | | | |  |  |
| Cellular permeabilities | PK-Sim Standard | | | | | |  |  |
| Weibull time (min) | 45 | 120 | | 120 | 10 | 60 | |  |
| Weibull shape | 0.92 | 0.92 | 0.92 | | 0.92 | 0.92 | |  |

^a^: The modeling parameters were built in the OSP library of PK-Sim.

^b,c^: The modeling parameters were taken from the reference S1 and S2.

The clinical observed PK data were taken from the references S1 (itraconazole), S3 (midazolam, rifampicin), S4 (ketoconazole) and S5 (posaconazole).

[S1] Chen Y, Ma F, Lu T, et al. Development of a physiologically based pharmacokinetic model for itraconazole pharmacokinetics and drug–drug interaction prediction[J]. Clinical pharmacokinetics, 2016, 55(6): 735-749.

[S2] Food and Drug Administration(FDA）：https://www.accessdata.fda.gov/drugsatfda_docs/nda/2015/208434Orig1s000ClinPharmR.pdf.

[S3] Hanke N, Frechen S, Moj D, et al. PBPK models for CYP3A4 and P‐gp DDI prediction: a modeling network of rifampicin, itraconazole, clarithromycin, midazolam, alfentanil, and digoxin[J]. CPT: pharmacometrics & systems pharmacology, 2018, 7(10): 647-659.

[S4] Daneshmend T K, Warnock D W. Clinical pharmacokinetics of ketoconazole [J]. Clinical pharmacokinetics, 1988, 14(1): 13-34.

[S5] Ezzet F, Wexler D, Courtney R, et al. Oral bioavailability of posaconazole in fasted healthy subjects: comparison between three regimens and basis for clinical dosage recommendations[J]. Clinical pharmacokinetics, 2005, 44: 211-220.

**Supplementary Table S2** The inhibition and induction parameters of modulators

| Modulators | K_i_ (μM) | EC_max_ | EC_50_ (μM) |
| --- | --- | --- | --- |
| Ketoconazole ^[S6]^ | 0.0038 (CYP3A4) | - | - |
|  | 2.23 (P-gp） | - | - |
| Rifampicin ^[S7,8]^ | - | 12.5(CYP3A4) | 0.25 |
|  | - | 10 (CYP2C8) | 0.12 |
|  | - | 0.34 (P-gp)^a^ | 2.5 |
| Posaconazole ^[S2]^ | 0.005(CYP3A4) | 5.2 (CYP3A4) | 0.07 |
| Itraconazole ^[S9]^ | 0.0013 (CYP3A4) | - | - |
| Hydroxy-itraconazole ^[S9]^ ^b^ | 0.0023 (CYP3A4) | - | - |

^a^: built in the PK-Sim^®^;^b^: metabolite of itraconazole.

[S6] Chen Y, Ma F, Lu T, et al. Development of a physiologically based pharmacokinetic model for itraconazole pharmacokinetics and drug–drug interaction prediction[J]. Clinical pharmacokinetics, 2016, 55(6): 735-749.

[S7] Xu Y, Zhou Y, Hayashi M, et al. Simulation of clinical drug-drug interactions from hepatocyte CYP3A4 induction data and its potential utility in trial designs[J]. Drug metabolism and disposition, 2011, 39(7): 1139-1148.

[S8] Dixit V, Moore A, Tsao H, et al. Application of micropatterned cocultured hepatocytes to evaluate the inductive potential and degradation rate of major xenobiotic metabolizing enzymes[J]. Drug Metabolism and Disposition, 2016, 44(2): 250-261.

[S9] Food and Drug Administration. (FDA):https://www.accessdata.fda.gov/drugsatfda_docs/nda/2017/

210259Orig1s000MultidisciplineR.pdf.

**Supplementary Table S3** The mean observed and predicted PK data for the substrate and modulators according to their respective PBPK model

| Drug | Parameters | Predicted | Observed | Predicted/Observed |
| --- | --- | --- | --- | --- |
| Midazolam（15 mg OD） | C_max_ (μg·mL^-1^) | 58.5 | 59.1 | 0.99 |
|  | AUC_0-48_ (μg·h·mL^-1^) | 246.5 | 243.2 | 1.01 |
|  | T_max_ (h) | 0.90 | 1.0 | 0.90 |
| Ketoconazole (400 mg OD) | C_max_ (μg·mL^-1^) | 7289.1 | 6240.0 | 1.17 |
|  | AUC_0-48_ (μg·h·mL^-1^) | 5235.9 | 4687.2 | 1.12 |
|  | T_max_ (h) | 1.7 | 1.5 | 1.13 |
| Itraconazole  (200 mg OD) | C_max_ (ng·mL^-1^) | 765.6 | 556.0 | 1.38 |
|  | AUC_0-48_ (ng·h·mL^-1^) | 5511.3 | 4500.5 | 1.22 |
|  | T_max_ (h) | 1.9 | 2.0 | 0.95 |
| Hydroxy- Itraconazole | C_max_ (ng·mL^-1^) | 723.4 | 639.0 | 1.13 |
|  | AUC_0-48_ (ng·h·mL^-1^) | 11676.6 | 13128.9 | 0.89 |
|  | T_max_ (h) | 3.5 | 4.0 | 0.88 |
| Rifampicin (600 mg OD) | C_max_ (ng·mL^-1^) | 10977.2 | 9540.0 | 1.15 |
|  | AUC_0-24_ (ng·h·mL^-1^) | 63191.4 | 62861.3 | 1.01 |
|  | T_max_ (h) | 1.2 | 1.5 | 0.80 |
| Posaconazole (800 mg OD) | C_max_ (μg·mL^-1^) | 146 | 123 | 1.19 |
|  | AUC_0-48_ (μg·h·mL^-1^) | 3796.2 | 3961.5 | 0.96 |
|  | T_max_ (h) | 8.5 | 6.0 | 1.42 |

**Supplementary Table S4** The PK alteration of midazolam concurrently with CRI and LOR, respectively.

| Drug | Parameters | Predicted ratios | Observed ratios |
| --- | --- | --- | --- |
| Midazolam（15 mg OD）  +CRI 300 mg BID | C_max_ (ng/mL) | 1.94 | 2.39 |
|  | AUC_0-inf_ (ng·h·mL^-1^) | 4.29 | 3.50 |
| Midazolam（2 mg OD）  +LOR 100 mg OD | C_max_ (ng/mL) | 0.50 | 0.60 |
|  | AUC_0-inf_ (ng·h·mL^-1^) | 0.66 | 0.37 |
