# Supplementary material for: Prediction of trough concentration and ALK occupancy in plasma and cerebrospinal fluid using physiologically based pharmacokinetic modeling of crizotinib, alectinib, and lorlatinib
Source: Front Pharmacol. 2023 Nov 22;14:1234262. doi: 10.3389/fphar.2023.1234262 (PMC10703149; doi:10.3389/fphar.2023.1234262)

**Figure S1 The mean predicted and observed plasma concentration-time profiles of midaolam (A), ketoconazole (B), rifampicin (C), posaconazole (D), and itraconazole (E) in healthy humans.** The blue squares (🞏) and red up-triangles (△) refer to the clinically observed pharmacokinetic data.
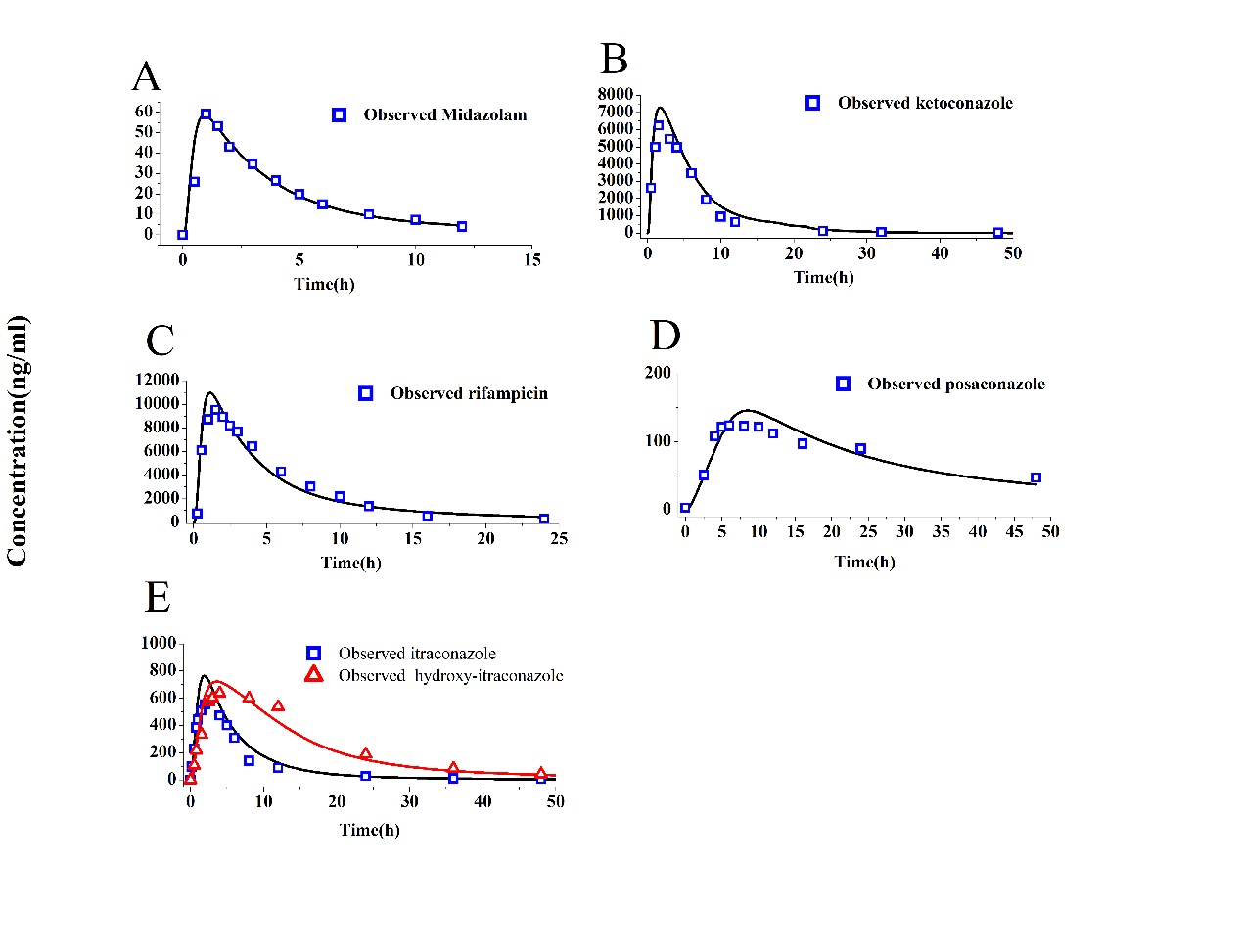

Supplement: Supplementary file 2 [file DataSheet1.docx]
